# Attenuation of intermittent hypoxia-induced apoptosis and fibrosis in pulmonary tissues via suppression of ER stress activation

**DOI:** 10.1186/s12890-020-1123-0

**Published:** 2020-04-16

**Authors:** Zhihui Shi, Linhao Xu, Hui Xie, Ruoyun Ouyang, Ya Ke, Rui Zhou, Wing-Ho Yung

**Affiliations:** 1 Department of Respiratory and Critical Care Medicine, The Second Xiangya Hospital, Central-South University, Changsha, China; 2 Research Unit of Respiratory Disease, Central-South University, Changsha, China; 3 School of Biomedical Sciences, Faculty of Medicine, The Chinese University of Hong Kong, Shatin, Hong Kong, SAR China; 4 Department of Cardiology, Affiliated Hangzhou First People’s Hospital, Zhejiang University School of Medicine, Hangzhou, China

**Keywords:** Obstructive sleep apnea, Endoplasmic reticulum stress, Intermittent hypoxia, Apoptosis, Fibrosis

## Abstract

**Background:**

Obstructive sleep apnea (OSA) is associated with pulmonary fibrosis and endothelial apoptosis in pulmonary tissues. Chronic intermittent hypoxia (IH) is considered to be the primary player in OSA, but the mechanisms underlying its effect on pulmonary tissues are unknown. Endoplasmic reticulum (ER) stress induced by IH treatment plays an important role in accelerating the process of fibrosis and induction of apoptosis.

**Methods:**

Mice were placed in IH chambers for 4 weeks with an oscillating oxygen (O_2_) concentration between 5 and 21%, cycling every 90s for 8 h daily. Mice were randomly divided into four groups: control group (normal oxygen), tauroursodeoxycholic acid (TUDCA) group (normal oxygen intraperitoneally injected with TUDCA), IH group and IH + TUDCA group. After 4 weeks, the proteins in three branch signaling pathways of ER stress, including protein kinase RNA (PKR)-like/Pancreatic ER kinase (PERK), activating transcription factor 6 (ATF-6) and inositol-requiring enzyme 1 (IRE-1), were evaluated. The cleaved caspase-3, caspase-12 and TUNNEL staining was assessed. Furthermore, the expression of transforming growth factor-β1 (TGF-β1) and thrombospondin-1(TSP-1), two extracellular matrix proteins that play critical role in fibrosis, were examined. Finally, Masson’s trichrome staining was performed to detect the expression of collagen.

**Results:**

After 4 weeks of IH treatment, the expressions of two ER stress markers, glucose regulated protein-78 (Grp78) and transcription factor C/EBP homologous protein (CHOP) were increased which was prevented by administration of the ER stress attenuator, TUDCA. The expressions of PERK, but not those of ATF-6 and IRE-1, were increased. The effects of IH were accompanied by an increased number of apoptotic cells and increased expressions of cleaved caspase-3 and caspase-12 in pulmonary tissues. In addition, histological examination suggested the presence of fibrosis after chronic IH treatment, indicated by increased expression of collagen, which was associated with the up-regulation of TGF-β1 and TSP-1 that are known to promote fibrosis. Similarly, TUDCA could reduce the extent of fibrotic area and the expression levels of these proteins.

**Conclusions:**

It reveals the roles of ER stress, especially the PERK pathway, in IH induced apoptosis and fibrosis in pulmonary tissues that might underlie the pulmonary complications observed in OSA.

## Background

Obstructive sleep apnea (OSA) is a common disorder characterized by repetitive collapse of the pharyngeal airway during sleep, resulting in intermittent hypoxia (IH) and reoxygenation. Several previous reports have indicated that the morphology and function of the lung are altered in OSA patients and IH animal models, including reductions in lung volumes and induction of pulmonary hypertension [1–3]. These symptoms were associated with pulmonary fibrosis and endothelial apoptosis [4–6]. According to previous studies, endoplasmic reticulum (ER) stress activation had been found in IH model and plays a critical role in apoptosis [7, 8].

It is well known that ER stress is caused by conditions that perturb the processing and folding of proteins, resulting in the accumulation of misfolded proteins [9]. Under IH condition, increased reactive oxygen species (ROS) production caused by the inhibition of complex I activity may lead to a loss of ER homeostasis and accumulation of misfolded proteins, and in turn activates ER stress [10, 11]. At the early stage of ER stress, unfolded protein response (UPR) was activated to enhance ER chaperone protein production, such as glucose regulated protein-78 (Grp78). This molecular chaperone could restore ER function via facilitating protein folding [12]. If the activation of ER stress is prolonged, ER stress-mediated apoptosis can be induced via three pathways, including protein kinase RNA (PKR)-like/Pancreatic ER kinase (PERK), activating transcription factor 6 (ATF-6) and inositol-requiring enzyme 1 (IRE-1) pathways [13]. These three pathways activate pro-apoptotic via the induction of the pro-apoptotic transcription factor C/EBP homologous protein (CHOP) [14, 15].

On the other hand, once ER stress is initiated, it is believed that fibrosis will be induced due to the acceleration of fibroblast proliferation and the expression of extracellular matrix protein, such as transforming growth factor-β1 (TGF-β1) and thrombospondin-1(TSP-1) [16, 17]. In the clinics, pulmonary fibrosis was noted in OSA patients [4, 18]. However, the major mechanisms underlying the effects of IH treatment on the pathological changes and dysfunction of pulmonary tissue are still largely unknown. We hypothesized that ER stress has a major contribution since inhibition of ER stress activation could potentially attenuate fibrosis and apoptosis [19].

At present, the most commonly used methods in the clinical treatment of OSA are surgery and continuous positive airway pressure (CPAP); however, each of these two methods has their own shortcomings and it is not effective for all patients [20, 21]. Therefore, there is an urgency in seeking a new therapeutic approach. For drug-based therapy, there have only been a few studies up to now. In order to identify precise and therefore better target sites for drugs, it is worth investigating the molecular mechanisms of pulmonary fibrosis and apoptosis in OSA.

Therefore, in order to investigate whether ER stress is present and its role in fibrosis and apoptosis in lung tissue after exposure to IH, tauroursodeoxycholic acid (TUDCA), a chemical chaperone that has been shown to reduce ER stress by facilitating proper protein folding and trafficking [22], was used to suppress ER stress activation and to observe the effect of reducing the number of apoptotic neurons and the progression of fibrosis.

## Materials and Methods

### Animals

Thirty-two C57BL/6 male mice (20–22 g) were purchased from the Animal Center of the Chinese University of Hong Kong (CUHK). The mice were kept in plastic cages under specific pathogen-free conditions with controlled lighting (12 h per day) and temperature (21 ± 2 °C). They were allowed free access to standard laboratory food and water at the animal laboratories of the CUHK. Four mice were placed in one cage. The procedures of experimentation were conducted with approval of the Animal Experimentation and Ethics Committee of the CUHK.

### Reagents

TUDCA was purchased from Sigma-Aldrich (St. Louis, MO). The BCA protein assay kit was purchased from Pierce Biotechnology (Rockford, USA). Antibodies, including glucose regulated protein-78 (Grp78, cat:3177), p-PERK (cat: 3179), activating transcription factor 4(ATF-4, cat:11815), phosphorylated eukaryotic initiation factor 2 alpha (p-elf2α, cat: 3398), caspase-3(cat: 9661) were purchased from Cell Signaling Technology (Beverly, MA). Caspase-12 (cat: ab62484) and transcription factor C/EBP homologous protein (CHOP cat: ab11419) were purchased from Abcam (Cambridge, USA). TUNEL kit was purchased from Millipore Corporation (Massachusetts, USA).

### Model of chronic intermittent hypoxia and TUDCA treatment

**Fig. 1:**
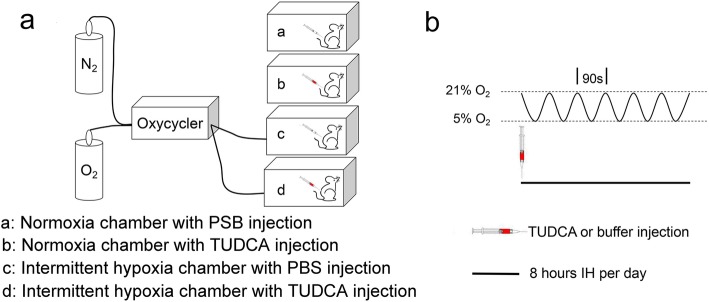
Schematics of the intermittent hypoxia (IH) model used in this study. **a** Conventional ventilated cages that mimic usual housing conditions are placed in computerized hypoxic chambers to achieve IH exposures in mice. The system is composed of the Oxycycler, which is responsible for gas (N_2_, O_2_), air-inlet regulation, gas tank (N_2_, O_2_), computer (not shown) and the intermittent hypoxia chamber. **b** The paradigm of intermittent hypoxia in OSA model. The concentration of oxygen (O_2_) was maintained between 5 and 21% cycling in every 90s

The protocol was well-accepted and utilized by other researchers to generate a sleep apnea model in rodent [8, 23–25]. Ordinary cages were placed in computer-controlled ventilation chambers (Oxycycler model A48XOV, Redfield, USA) to achieve IH exposures in the animals. The concentration of oxygen (O_2_) was maintained between 5 and 21%, cycling every 90s for 8 h (8:00 A.M. to 4:00 P.M. Fig. 1). Control animals were exposed to alternating periods of room air in identical chambers. The intermittent hypoxia treatment lasted for 4 weeks.

According to our previous study [8], TUDCA dissolved in phosphate buffer saline (PBS) was intra-peritoneally injected daily (100 mg/kg) at 7:30 AM to 8:00 AM before IH treatment. The control group received PBS alone. The mice were randomly assigned into four groups: PBS-treated group (control group), TUDCA-treated group (TUDCA group), PBS-treated IH group (IH group) and TUDCA-treated IH group (IH + TUDCA group).

### Tissue processing

After 4 weeks of IH exposure, the mice were deeply anesthetized with pentobarbital sodium salt (Sigma-Aldrich, Darmstadt, Germany) at the dosage of 30 mg/kg by intraperitoneal injection. Then, four mice of each group were decapitated to obtain pulmonary tissues (upper lobe of left lung), which were stored at − 70 °C for Western blot analysis and quantitative real-time RT-PCR analysis. The thoracic cavities of another four mice in each group were opened by surgical scissors and perfused with 30–40 ml fixative containing 4% paraformaldehyde in 0.1 M phosphate buffer (pH 7.4) through the cardiac aorta. After perfusion, the left lung was removed. The tissue was post-fixed in the same fixative for 6 h and embedded in paraffin, and cut into 4 μm sections.

### Western blot analysis

Proteins were obtained from pulmonary tissue, which was homogenized with ice-cold radioimmunoprecipitation assay buffer containing 1 mM of PMSF and phosphatase inhibitor cocktail (Roche, Germany). BCA Protein Assay Reagent Kit (Pierce Biotechnology, Rockford, USA) was used to measure the protein concentration. Protein was separated by 12% sodium dodecyl sulfate-polyacrylamide gel electrophoresis and transferred to a PVDF membrane (Millipore Corporation; Billerica, MA, USA). Non-fat dry milk (5%) was used to block the menbranes. The blocked membranes were then incubated with the primary antibody (Grp-78, p-PERK, ATF-4, p-elf2α, caspase-3, caspase-12 and CHOP) at 4 °C overnight. After stringent washes with Tris-buffered saline containing 0.1% Tween-20 (TBST), blots were incubated with a fluorescent secondary antibody (LI − COR Biotechnology, Lincoln, USA) for 1 h at room temperature. An Odyssey scanner was used to detect the intensities of the specific bands (LI − COR Biotechnology, Lincoln, USA). Equal amount of target protein was confirmed by β-actin antibody (Bio-Rad Laboratories, California, USA).

### Histological staining

Paraffin sections were rinsed in 0.01 M PBS and mounted onto 0.02% poly-L-lysine-coated slides. The expression of collagen was detected by Masson’s trichrome staining which stains cell nuclei, cytoplasm, and collagen in dark brown, red and blue color respectively.

Section was firstly stained by Weigert’s working hematoxylin for 10 min. After washing with distilled water, they were then stained with Biebrich scarlet for 5 min. Finally, the sections were treated with phosphomolybdic acid and differentiated by aniline blue. After staining, the sections were dehydrated with absolute alcohol and xylene, then cover-slipped with Permount, and the sections were observed under a light mi croscope (Zeiss Microscope Axiophot 2) by an investigator blind to the treatment. Three or four slices from each mouse were quantifed and a total of 15 slices from four mice in each group were analyzed. The area of collagen expression was automatically identified by Metamorph 7.5 software with the function of colocalization and deconvolution (Molecular Devices, USA).

### Quantitative real-time RT-PCR analysis

Total RNA was isolated from pulmonary tissue homogenates using TRIzol reagent (Invitrogen, USA). The RNA concentration was measured by NanoDrop (Thermo,USA) and complementary DNA (cDNA) was synthesized using a Transcriptor First Strand cDNA Synthesis Kit (TaKaRa, Japan). For real-time qPCR, cDNA was firstly denatured by heating to 94 °C for 3 min and amplified by 40 cycles of PCR (denaturation at 94 °C for 15 s, annealing at 60 °C for 1 min, and extension at 72 °C for 30 s). A single production was confirmed by the dissociation curve. PCR primers (Invitrogen, USA) used in this study are as follows:

Grp-78: (forward: 5′-GGTGCAGCAGGACATCAAGTT-3′;

reverse: 5′-CCCACCTCCAATATCAACTTGA-3′);

CHOP: (forward: 5′-CTGCCTTTCACCTTGGAGAC-3′;

reverse: 5′- CGTTTCCTGGGGATGAGATA - 3′);

Frameshift splice X box-binding protein 1 (XBP1-s): (forward: 5′ -AAGAACACGCTTGGGAATGG-3′;

reverse: 5′- ACTCCCCTTGGCCTCCAC -3′);

ATF4: (forward: 5′ -GCAGCAGCACCAGGCTCT-3′;

reverse: 5′-TTGTCCGTTACAGCAACA CTG-3′);

TSP-1: (forward: 5′-CACCTCTCCGGGTTACTGAG-3′;

reverse: 5′-GCAACAGGAACAGGACACCTA-3′);

TGF-β1: (forward: 5′-CCGCAACAACGCCATCTATG-3′;

reverse: 5′-CTCTGCACGGGACAGCAAT-3′);

β-actin: (forward: 5′-ACCCACACTGTGCCCATCTA-3′;

reverse: 5′- CACGCTCGGTCAGGATCTTC-3′).

The results were calculated by the 2-^△△^Ct method based on previous study [7].

### TUNEL staining

Apoptosis was detected by in situ terminal transferase mediated dUTP nick end labeling (TUNEL technique), using an ApopTag1 kit (Millipore Corporation, MA) and following the instructions of the manufacturer. First, the section (5 μm thick) of pulmonary tissue were incubated with proteinase-K (20 μg/ml in PBS) for 15 min at room temperature. Second, the sections were washed by PBS and then quenched in 3% H_2_O_2_ in PBS for 5 min.. Third, they were then treated with biotin–deoxyuridine triphosphate in the working solution of deoxynucleotidyl transferase for 1 h in a humidified chamber at 37 °C. Fouth, the reaction was stopped with buffer solution at room temperature. The sections were then rinsed with PBS for three times, and anti-digoxigenin-peroxidase were applied and incubated for 30 min at room temperature. Finally, the sections were reacted with 0.05% diaminobenzidine with 0.01% H_2_O_2,_ and counterstained in 0.5% methyl green. After processing and dehydration, the slides were mounted with Permount and observed under a light microscope (Zeiss Microscope Axiophot 2, USA). Three or four slices from each mouse were quantified and a total of 15 slices from four mice in each group were analyzed.

### Statistical analysis

Data are presented as means ± standard error of means. The results were analyzed by two-way ANOVA followed by a Newman-Keuls post hoc test for multiple comparisons. Difference with *P*-value less that 0.05 was considered significant.

## Results

### Chronic IH induces ER stress in pulmonary tissue

**Fig. 2:**
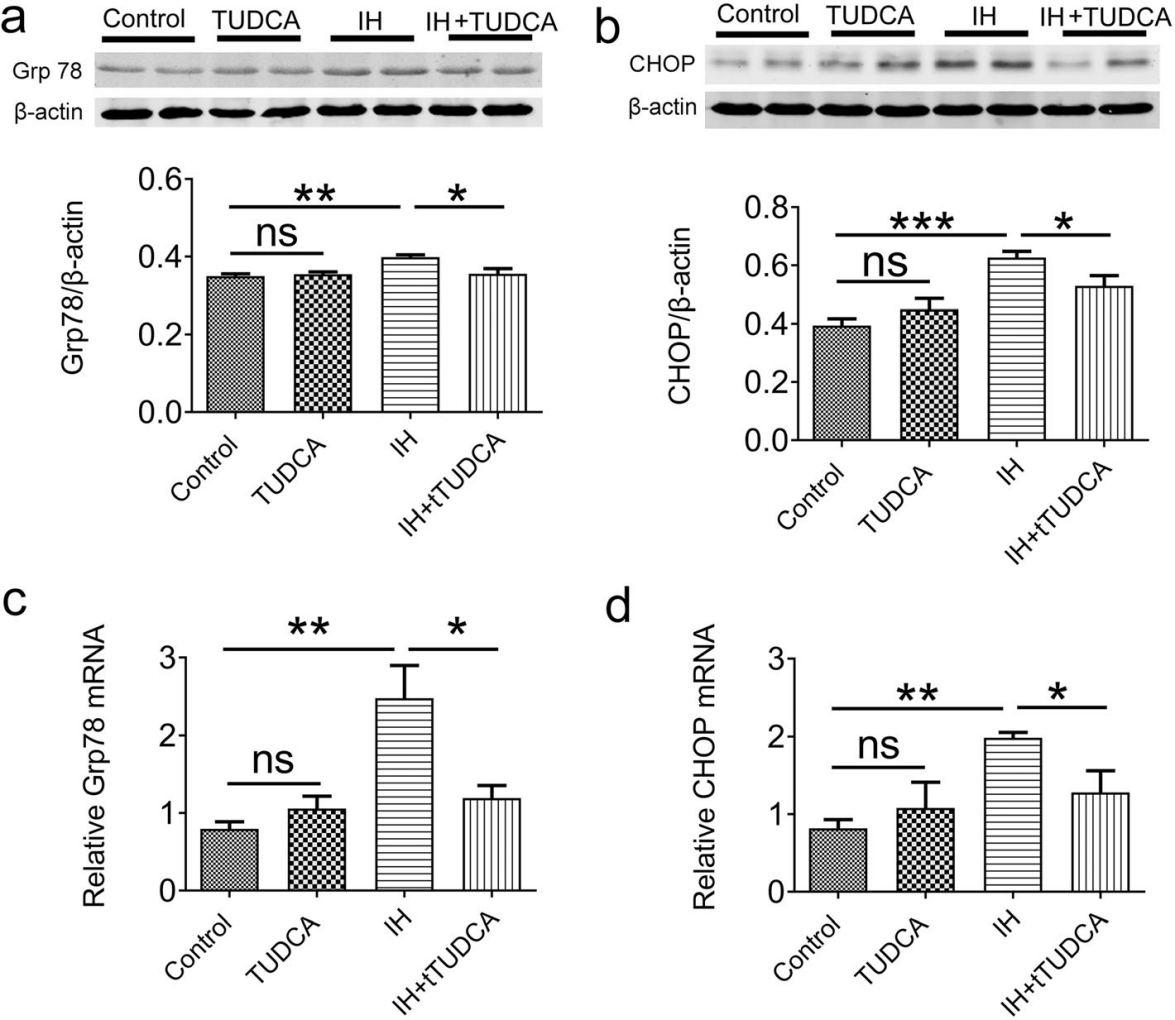
ER stress activation in pulmonary tissue after exposure to 4 weeks of intermittent hypoxia (IH). The expression levels of Grp78 (**a**) and CHOP (**b**) in whole pulmonary tissue homogenates were detected by Western blot. Grp78 and CHOP were upregulated after 4 weeks of IH treatment, which were prevented by the injection of TUDCA (*n* = 4). The expression of Grp78 (**c**) and CHOP (**d**) mRNA in pulmonary tissue was increased after 4 weeks of IH treatment (*n* = 4).. *P < 0.05; **P < 0.01; ***P < 0.001; ns *P* > 0.05

To test whether ER stress could be triggered in pulmonary tissue by chronic IH, mice were subject to 4 weeks of IH exposure, which consisted of daily 8 h of fluctuating O2 level between 21 and 5% in every 90s in the ambient environment (see Materials and Methods and Fig. 1). Western blot and real-time PCR were used to explore the protein and gene expression levels respectively of Grp78 and CHOP. As shown in Fig. 2a, after 4 weeks of IH treatment, two-way ANOVA disclosed that both TUDCA administration and IH treatment had significantly effects on the protein level of Grp78 [TUDCA administration: F (1,12) = 5.10, *P* = 0.0433; IH treatment: F (1,12) = 8.91, *P* = 0.0114]. However, two-way ANOVA confirmed that only IH treatment had a significant effect on the protein level of CHOP [TUDCA administration: F (1,12) = 0.47, *P* = 0.5046; IH treatment: F (1,12) = 26.57, *P* < 0.001; Fig. 2b]. Post hoc Newman-Keuls revealed that while administration of the ER stress inhibitor TUDCA had no effect on the expression levels of these two molecules in naïve animals, co-treatment of TUDCA during IH exposure could significantly suppress the increase of these two molecues when compared with control group (*P* < 0.01, Fig. 2a and b). At the same time, the mRNA levels of Grp78 and CHOP were significantly affected after 4 weeks of IH treatment by two-way ANOVA analysis [F (1,12) = 10.89, *P* = 0.0063 for the expression of Grp78 mRNA; F (1,12) = 11,14, *P* = 0.0059 for the expression of CHOP, Fig. 2c and d]. However, TUDCA administration did not affect these two mRNA levels [F (1,12) = 2.12, *P* = 0.1713 for Grp78 mRNA; F (1,12) = 3.24; *P* = 0.0971 for CHOP mRNA]. Post hoc Newman-Keuls revealed TUDCA treatment could suppress increased Grp78 and CHOP mRNA expression induced by IH (*P* < 0.05, Fig. 2c and d). These findings confirm that chronic IH exposure activates ER stress in pulmonary tissue.

### IH-induced ER stress is dependent on the PERK but not the IRE1 and ATF6 pathways

**Fig. 3:**
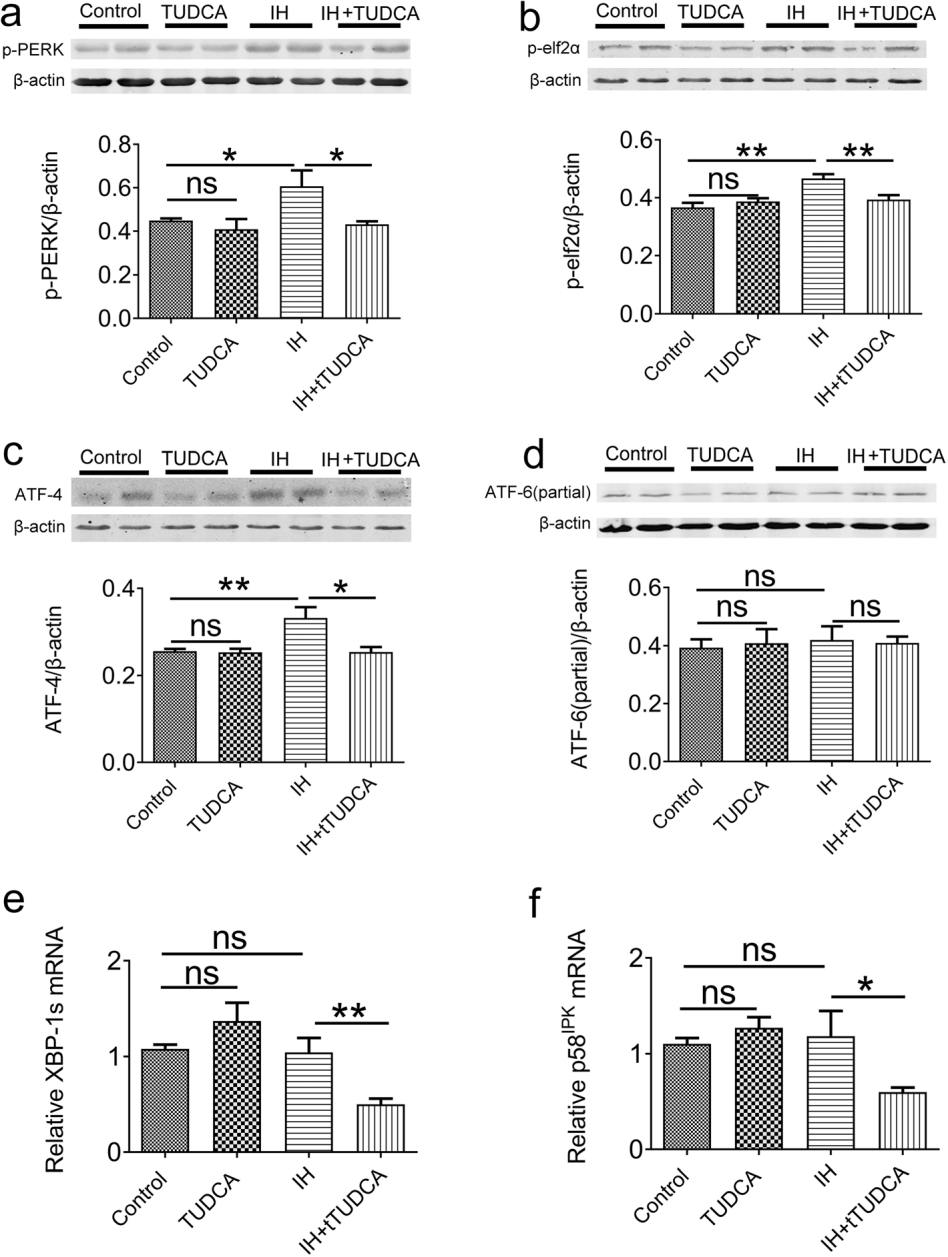
ER stress activation is dependent on PERK pathway, but not IRE1 and ATF6 pathways. The protein levels of p-PERK (**a**), p-eIF2a (**b**) and ATF4 (**c**) were elevated in pulmonary tissue (*n* = 4). **d** The expression of active ATF6 was not altered after exposure to 4 weeks of IH. The expression of XBP-1 s (**e**) and p58^IPK^ (**f**) mRNA also did not change (*n* = 4). *P < 0.05; **P < 0.01; ns *P* > 0.05

To dissect the exact pathways leading to ER stress in pulmonary tissue, we examined the protein or gene expression levels of specific molecules in these different pathways. First, a number of proteins involved in the PERK pathways were assessed. As shown in Fig. 3a-c, compared with the control, two-way ANOVA revealed that TUDCA could affect the expression of p-PERK, ATF-4, but not p-elf2α[F (1, 12) = 5.79, *P* = 0.0331 for p-PERK; F (1,12) = 7.56, *P* = 0.0176 for ATF-4; F (1,12) = 3.19, *P* = 0.0995 for p-elf2α], meanwhile, IH treatment had a significant effect on the expression of p-elf2α and ATF-4 [F (1,12) = 12.9, *P* = 0.0036 for p-elf2α; F (1,12) = 6.81, *P* = 0.0228 for ATF-4; F (1,12) = 4.10, *P* = 0.0659 for p-PERK]. Post hoc Newman-Keuls revealed the expression of p-PERK, ATF-4 and p-elf2α were significantly increased after IH exposure, which were rectified by TUDCA treatment (*P* < 0.01). In contrast, the level of ATF6 was not affected by TUDCA or IH treatment [TUDCA: F (1,12) = 0.006, *P* = 0.9383; IH: F (1,12) = 0.13, *P* = 0.7224, Fig. 2d]. For the IRE1 pathway, the mRNA levels of XBP-1 s were also altered by IH application [TUDCA: F (1,12) = 0.31, *P* = 0.5897; IH: F (1,12) = 18.91, *P* < 0.001], However, the mRNA levels of p58^IPK^ was not affected by TUDCA and IH [TUDCA:F (1,12) = 1.96, *P* = 0.1871; IH: F (1,12) = 3.94, *P* = 0.07]. Post hoc Newman–Keuls’s test indicated that these two mRNA levels were unaffected in IH group when compared with control group (*P* > 0.05, Fig. 3e and f). The only significant effect that we observed was a decrease in XBP-1 s and p58^IPK^ level in the IH group in the presence of TUDCA when compared with IH group (*P* < 0.05, Fig. 3e and f). Together, these results indicate that IH-induced ER stress in pulmonary tissue is dependent specifically on the PERK pathway but not the IRE1 and ATF6 pathways.

### ER stress inhibitor reduces IH-induced cell death by suppressing apoptosis

**Fig. 4:**
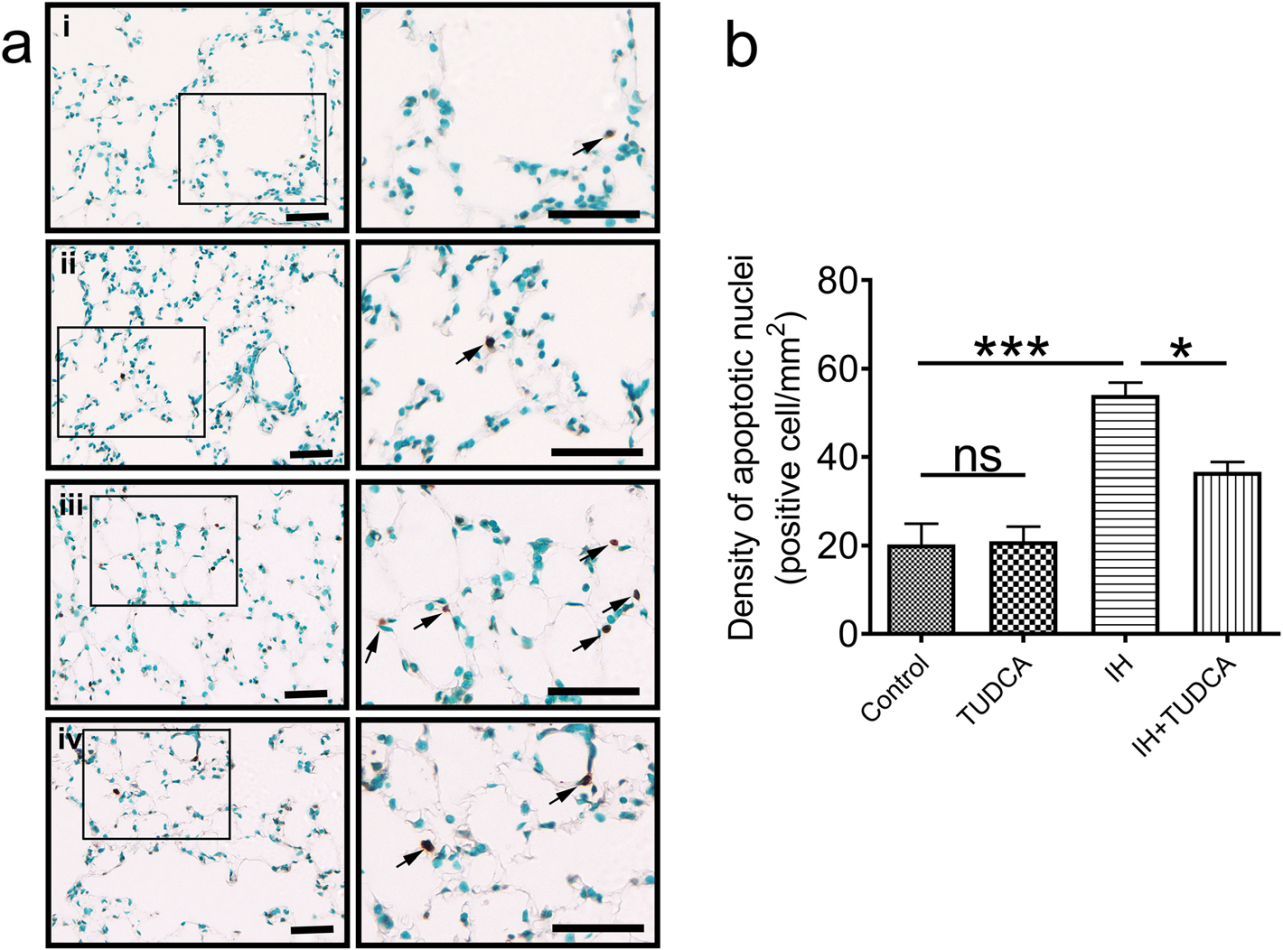
ER stress induced cell death after exposure to chronic IH. **a** A small number of apoptotic cells were observed around pulmonary alveoli in the control normoxia group (i) and TUDCA treatment (ii). Significantly increased number of apoptotic alveolar cells were observed in the IH group (iii); however, apoptotic cell counts were lower in the IH+ TUDCA group when compared to the IH group. The inset of each picture is enlarged and displayed on the right. Most of the apoptotic alveolar cells (indicated by arrowheads) were type II alveolar epithelial cells. The pooled data from 4 mice for each group are summarized in b (*n* = 4). Scale bar: 25 μm. *P < 0.05; **P < 0.01; ns *P* > 0.05

In order to investigate whether ER stress induced apoptosis in pulmonary tissue after 4 weeks of IH exposure, we conducted histological analysis to quantify TUNEL-positive cells. Two-way ANOVA revealed that apoptotic cells which was dyed brown was both affected by TUDCA and IH [TUDCA: F (1,12) = 5.96, *P* = 0.0311; IH:F (1,12) = 52.07, *P* < 0.001]. Post hoc Newman-Keuls test indicated that the density of apoptotic cells was much higher in the IH group (54.07 ± 2.85.7/mm^2^, Fig. 4aiii) when compared with the control (20.28 ± 4.73/mm^2^, Fig. 4ai,, *P* < 0.001) and TUDCA groups (21.00 ± 3.36/mm^2^, Fig. 4aii,, *P* < 0.001) suggesting increased apoptosis induced by IH treatment. However, co-treatment with TUDCA during IH could significantly reduce the level of apoptosis (36.64 ± 2.28/mm^2^, Fig. 4aiv, post hoc Newman-Keuls *P* < 0.05).

**Fig. 5:**
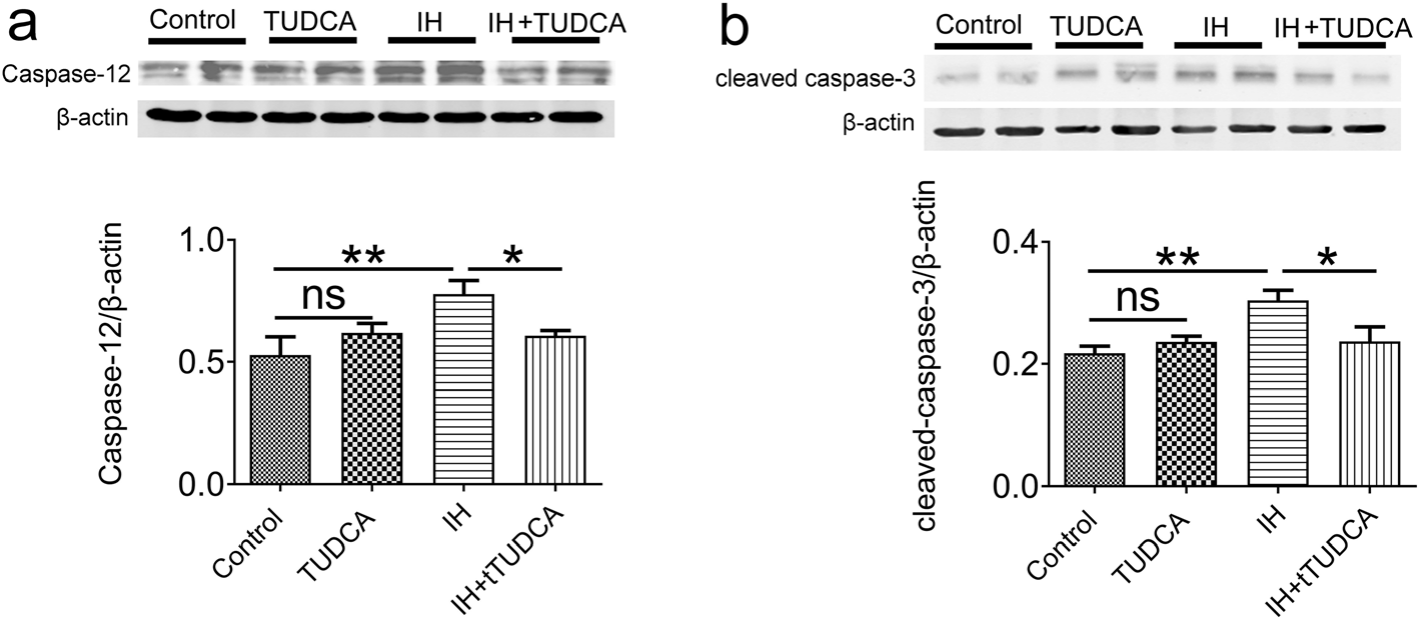
Western blot analysis of caspase-12 and cleaved caspase-3 in pulmonary tissue homogenates in cchronic IH. Caspase-12 (**a**) and cleaved caspase-3 (**b**) were both up-regulated after 4 weeks of IH treatment. Western blot bands were normalized to β-actin. These effects were prevented by administration of TUDCA. (*n* = 4) . *P < 0.05; **P < 0.01; ns *P* > 0.05

We further confirmed the involvement of ER stress in inducing apoptosis in pulmonary tissue by examining the expression of caspase-12, a specific molecular marker of apoptosis as a result of ER stress, as well as cleaved caspase-3, an important protein marker of apoptosis. As shown in Fig. 5, two-way ANOVA indicated that the expression of caspase-12 and cleaved caspase-3 were affected by IH treatment [F (1,12) = 5.30, *P* = 0.04 for caspase-12; F (1,12) = 6.96, *P* = 0.0217 for cleaved caspsae-3], but not TUDCA [F (1,12) = 0.60, *P* = 0.4548 for caspase-12; F (1,12) = 2.18, *P* = 0.1653 for cleaved caspsae-3]. Post hoc Newman-Keuls test indicated that the protein levels of these two markers were significantly increased by chronic IH treatment, which were suppressed by TUDCA treatment (*P* < 0.05, Fig. 5).

### ER stress activates TGF-β/TSP-1 pathway-dependent fibrosis in chronic IH

**Fig. 6:**
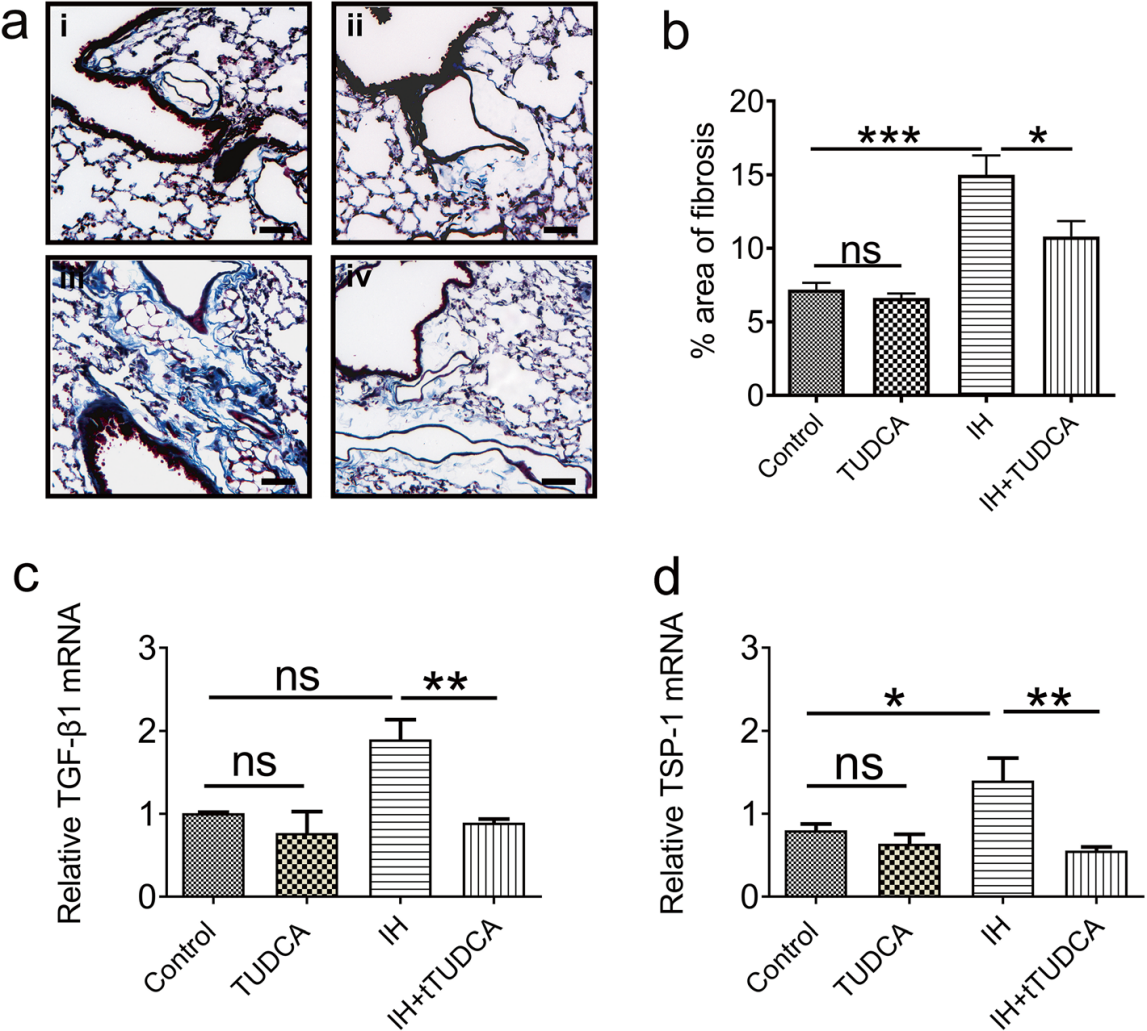
TUDCA attenuates the progression of fibrosis inpulmonary tissue via suppressing TSP-1/TGF-β1 pathway. **a** Representative micrographs were obtained from (i) the control group; (ii) TUDCA group; (iii) IH group; and (iv) IH+ TUDCA group. The expression of pulmonary fibrosis was identified using Masson’s trichrome staining. The bright blue color represents the distribution of collagen. Scale bars: 50 μm. **b** The percent area of collagen was significantly increased after 4 weeks of IH exposure, which was reduced by TUDCA application (*n* = 4). **c**-**d** mRNA expressions of TGF-β1(**c**) and TSP-1 (**d**) in pulmonary tissue. Analysis of the mRNA expressions reveals that TGF-β1and TSP-1mRNA were significantly elevated in the IH group when compared with the control and TUDCA groups, but prevented by the application of TUDCA (*n* = 4). *P < 0.05; **P < 0.01; ***P < 0.001; ns *P* > 0.05

To examine if pulmonary fibrosis is indeed a consequence of ER stress induced by chronic IH, we used Masson’s trichrome staining to detect the expression of collagen. As shown in Fig. 6a, a few blue spots of stained collagen were seen around the pulmonary alveoli, and on average occupying 7.18 ± 0.47% and 6.61 ± 0.31% of areas in the control and TUDCA group (Fig. 6ai and ii). However, increased expression of collagen was observed in the pulmonary alveoli and alveolar of IH group, occupying 15.01 ± 1.32% of area (Fig. 6aiii) and suggesting fibrosis 6. Two-way ANOVA analysis of the percentage of area of fibrosis revealed significant alternation of collagen expression [TUDCA: F (1,12) = 7.24, *P* = 0.0196; IH: F (1,12) = 45.73, *P* < 0.01]. Meanwhile, the expression of collagen was significantly reduced by TUDCA application when compared with the IH group occupying 10.08 ± 1.05% of area (Fig. 6aiv, post hoc Newman-Keuls, *P* < 0.05,), indicating that the level of fibrosis was attenuated.

In order to gain insight into the mechanism of TUDCA on reducing collagen, some extracellular matrix proteins, including transforming growth factor-β1 (TGF-β1) and thrombospondin-1 (TSP-1), were measured. Indeed, two-way ANOVA revealed that the mRNA levels of TGF-β1 and TSP-1 were only altered by TUDCA [F (1,12) = 12.42, *P* = 0.0042 for the mRNA level of TGF-β; F (1,12) = 10.68, *P* = 0.0067 for the mRNA level of TSP-1]. Furthermore, post hoc Newman–Keuls’s test indicated that the mRNA levels of TGF-β and TSP-1 were both elevated in the IH group when compared with control group (*P* < 0.05, Fig. 6c and d), and TUDCA could reduce the expression of these mRNAs (*P* < 0.05, Fig. 6c and d). These findings suggest that ER stress-induced fibrosis is triggered in our model and the ER stress inhibitor TUDCA could substantially attenuate the fibrosis level via the TGF-β/TSP-1 pathway.

## Discussion

Previous studies hinted that ER stress could be activated in some organs under chronic IH, such as the brain and myocardium [7, 8]. However, whether ER stress is induced in lung tissue under the same condition is unknown. This study is the first to observe ER stress activation in pulmonary tissue in an animal model of IH. We found that ER stress-induced apoptosis and fibrosis were present after 4 week’s IH exposure, which could be abolished by TUDCA, a common and widely used ER stress inhibitor.

The endoplasmic reticulum (ER) is an important organelle which modulates protein biosynthesis, folding, lipid biosynthesis, apoptosis and calcium homeostasis. Once the homeostasis is upset under cellular stress conditions such as hypoxia, accumulation of unfolded and misfolded proteins in the ER could lead to ER stress. When ER stress is triggered, increased Grp78 is dissociated from PERK, IRE1 and ATF6 to bind with the unfolded or misfolded protein. In our study, the expression of Grp78 was indeed increased after exposure to 4 weeks of IH treatment. This is accompanied by elevated expression of CHOP which is well known to promote cell death. TUDCA treatment could suppress the expression of these two proteins, which was consistent with our previous study that TUDCA could suppress ER stress activation via decreasing the expression of Grp78 and CHOP [8]. These pieces of evidence together suggest that chronic IH treatment could activate ER stress in pulmonary tissue.

It is commonly accepted that the PERK, ATF-6 and IRE1 pathways are all capable of inducing the expression of CHOP [14, 15]. First, PERK phosphorylates eukaryotic initiation factor 2 alpha (elf2α), which leads to regulation of the translation of several genes. The most studied of these genes is ATF4, which encodes CHOP transcription. Second, active ATF6 then moves to the nucleus and induces genes with an ER stress response element in their promoter, including CHOP and X box-binding protein 1 (XBP-1). Finally, the endonuclease activity of IRE1 removes a 26-nucleotide intron from the XBP1 mRNA previously induced by ATF6, to generate frameshift splice variant (XBP-1 s) which has diverse targets such as p58^IPK^, and finally activates the expression of CHOP [15]. In our results, it was shown that the expression levels of p-PERK, p-elf2 and ATF-4 were all enhanced after IH-induced treatment; however, the expressions of XBP-1 s, p58^IPK^ and active ATF6 were not elevated, suggesting that the IRE1α–XBP1 and ATF6 pathways are probably not involved. Therefore, the increased CHOP expression was due to the activation of the PERK pathway, but not the IRE1 and ATF6 pathways in our model. One potential explanation is that, according to some previous studies, the IRE1α–XBP1 and ATF6 pathways are turned off in cells undergoing prolonged ER stress, whereas PERK signaling is sustained in the pro-apoptotic phase [26–28]. Although studies on the mechanisms of IRE1–XBP1 and ATF6 pathways deactivation are sparse, IRE1–XBP1 and ATF6 pathways were reported to promote cell survival instead of inducing cell death under the condition of chronic ER stress [14, 27]. In contrast, PERK signaling was found to inhibit translation and induce pro-apoptotic transcription regulator CHOP [27, 28], which is consistent with our results. It is noteworthy that a previous study reported that IH exposure decreases rather than increases ER stress markers [29]. This disparity could be due to that the duration of IH treatment in this study was only 3 days which is quite different from our model. In fact, from our previous study, we did not observe ER stress activation in the hippocampus after 7 days of IH treatment, but was significantly activated after 14 days of IH treatment [8]. Also, it has been reported that PERK-elf2-ATF-4 arm is activated to induce apoptosis in prolonged IH treatment [30]. Caspase-12 has been proposed as a key mediator under the execution phase of ER stress-induced apoptosis, with its activation by means of excessive ER stress and in turn activating pro-caspase-3, leading to apoptosis [31]. We observed that the expression levels of caspase-12 and cleaved caspase-3 were significantly elevated in the IH group, while the number of TUNEL-positive alveolar epithelial cells increased, which could be suppressed by TUDCA treatment. Apoptosis has a critical role in lung function, since it has been found that increased apoptotic cell count could lead to lung dysfunction in LPS-induced inflammatory models. Furthermore, reduction of apoptotic cells by saturated hydrogen saline could improve oxygenation and rescue abnormal pulmonary structure [32]. Although there are no previous studies demonstrating that apoptosis is present in pulmonary tissues of OSA patients and IH animal models, a large amount of free DNA was found in the serum of OSA patients implicating the presence of apoptosis [33]. In this work, we demonstrated that apoptosis was indeed activated in pulmonary tissue of the IH model which was induced by ER stress activation.

In addition to apoptosis, ER stress activation plays a critical role in the development of pulmonary fibrosis [34, 35]. In the clinics, it was found that idiopathic pulmonary fibrosis is a characteristic of OSA patients [18]. It is well known that increased collagen deposition and synthesis are critical contributors of fibrosis [36]. In this study, we observed that the expression of collagen as detected by Masson trichrome staining was elevated in the IH group. At the same time, TUDCA application could reduce collagen expression, which was consistent with a previous study [37]. Although the precise mechanism of ER stress-induced fibrosis is not fully elucidated, some investigators suggested that epithelial mesenchymal transition (EMT) induced by ER stress contributes to fibrosis [34]. According to previous study, it has been shown that pharmacological inhibition of ER stress prevents transforming growth factor β-1 (TGF-β1)-inducted EMT features [35]. TGF-β1 is a well-known profibrotic cytokine that activates fibroblasts and leads to tissue fibrosis [38]. TSP-1, a multifunctional glycoprotein, is a major activator of TGF-β1 [39]. In this study, the results of the real time PCR revealed that TGF-β1 and TSP-1 mRNAs were increased after exposure to 4 weeks of IH treatment and reduced by administration of TUDCA. Although the underlying mechanism was not revealed in this study, we speculate that PERK-elf2-ATF-4-CHOP signaling pathway plays an important role since only PERK signaling pathway was activated in our model. A large number of studies have shown that interference or knockout of CHOP could significantly decrease the production of TGF-β1 [40, 41], which could be associated with suppressing NF-κB signaling [39] since NF-κB is a major regulator to modulate the expression of TSP-1 and TGF-β1 [42, 43].

It is well known that NF-κB is a common proinflammatory transcription factor and was activated in OSA patients [44]. Indeed, OSA appears to have an inflammatory component. In addition to NF-κB activation, many proinflammatory cytokine, such as tumor necrosis factor-α (TNF-α), interleukin-1 beta (IL-1β) and interleukin-6 (IL-6), are all found in high concentrations in OSA patients [45]. However, the participation of this mechanism has not yet been clarified. Some researchers have found that an increase in ROS production under IH cycles could induce inflammatory pathways that activate multiple proinflammatory cytokines [46]. Under conditions of ER stress, additional misfolded or unfolded proteins are synthesized and known to generate additional ROS as a byproduct [47]. Also, according to our previous study, TUDCA could reduce the production of ROS [8]. Therefore, the effect of TUDCA on attenuating fibrosis might be associated with suppressing inflammation and ROS production. Finally, as shown in this study, apoptosis and fibrosis in the lung are two consequences of IH treatment. Whether there is a causal relationship between these two phenomena would need further investigation.

## Conclusion

In summary, the present study demonstrates that ER stress is activated, and could be a major factor, in the pathogenesis of IH-induced apoptosis and fibrosis in pulmonary tissues that might underlie pulmonary complications observed in OSA. TUDCA, a well-known ER chemical chaperone, inhibits PERK pathway-dependent ER stress activation in our model. Moreover, TUDCA attenuates IH-induced pulmonary fibrosis by suppression of the TSP-1/TGF-β1 pathway. Thus, chemical chaperones may have potential preventive and therapeutic uses for protecting against pulmonary apoptosis and fibrosis in IH models.

## Data Availability

The data that support the findings of this study are available from School of Biomedical Sciences, the Chinese university of Hong Kong. The datasets used and analyzed during the current study are available from the corresponding author (Wing-Ho Yung) on reasonable request.

## Abbreviations

ATF-4: Activating transcription factor 4
ATF-6: Activating transcription factor 6
CHOP: Transcription factor C/EBP homologous protein
EMT: Epithelial mesenchymal transition
CPAP: Continuous positive airway pressure
ER: Endoplasmic reticulum
IH: Intermittent hypoxia
IRE-1: Inositol-requiring enzyme 1
IL-1β: Interleukin-1 beta
IL-6: Interleukin-6
Grp78: Glucose regulated protein-78
OSA: Obstructive sleep apnea
p-elf2α: Phosphorylated eukaryotic initiation factor 2 alpha
PERK: Protein kinase RNA (PKR)-like/Pancreatic ER kinase
ROS: Reactive oxygen species
UPR: Unfold protein response
TGF-β1: Transforming growth factor-β1
TNF-α: Tumor necrosis factor-α
TSP-1: Thrombospondin-1
TUDCA: Tauroursodeoxycholic acid
XBP-1: X box-binding protein 1
